# Genomic features of the methylosome protein MEP50 and its implications in hormone signaling and cancer

**DOI:** 10.1530/EC-25-0444

**Published:** 2025-09-24

**Authors:** Gareth Pollin, Young-In Chi, Raul Urrutia, Gwen Lomberk

**Affiliations:** ^1^ Linda T. and John A. Mellowes Center for Genomic Sciences and Precision Medicine (Mellowes Center), Medical College of Wisconsin, Milwaukee, Wisconsin, USA; ^2^ Division of Research, Department of Surgery, Medical College of Wisconsin, Milwaukee, Wisconsin, USA; ^3^ Department of Biochemistry, Medical College of Wisconsin, Milwaukee, Wisconsin, USA; ^4^ Department of Pharmacology and Toxicology, Medical College of Wisconsin, Milwaukee, Wisconsin, USA

**Keywords:** functional genomics, methylosome, MEP50, WDR77, alternative splicing

## Abstract

**Graphical Abstract:**

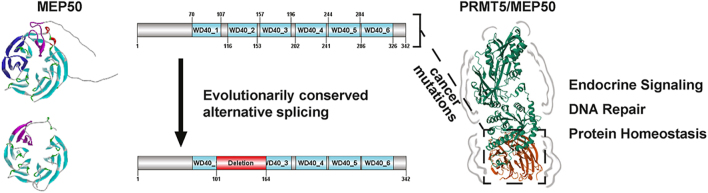

**Abstract:**

Dysfunction of several WD40 family proteins causes diverse endocrine diseases. Until recently, MEP50, a WD40 protein, was considered a gene of unknown significance because no inherited disease had been linked to its function. However, genetic inactivation of MEP50 in mouse models or somatic mutations in humans drives oncogenesis in several endocrine-related cancers, including those of the prostate, breast, and uterus. In this study, we generate new knowledge through a multi-tier integration of evolutionary genomics, sequence and structural analyses, molecular mechanic calculations, and dynamic simulations of wild-type and cancer-mutated MEP50 proteins. Indeed, we find that a conserved splicing event across evolution generates an alternative MEP50 isoform, which is smaller than the canonical MEP50 and lacks the final β-sheet of the first WD40 domain, the entirety of the second WD40 domain, and the first β-sheet of the third WD40 domain. Notably, we find that this novel, short MEP50 (s-MEP50) transcript encodes a 278 amino acid protein that retains aspects of the key regulatory and interaction sites, including those critical for androgen receptor and PRMT5 binding. Finally, we analyze the mutational landscape of MEP50 in endocrine-regulated cancers and use molecular mechanic calculations and dynamic simulations to reveal that cancer-associated mutations disrupt conserved bonding networks and induce widespread structural destabilization within the WD40 domain architecture. Thus, by combining evolutionary, structural, and biophysical approaches, we advance the understanding of MEP50 genomics, providing significant mechanistic and clinically relevant insights into endocrine-regulated tissues and their cancers.

## Introduction

The WD repeat-containing protein 77 (MEP50/WDR77) is a multifunctional scaffolding protein within endocrine signaling cascades and serves as a core structural component of the PRMT5 methylosome complex, which incorporates one of several mutually exclusive adaptor proteins, including RIOK1, COPRS, or pICLn (1, 2, 3). MEP50 employs its WD40 β-propeller architecture to mediate ligand-dependent transcriptional activation of steroid nuclear receptors. Structural analyses of MEP50 demonstrate that its seven-bladed β-propeller, formed by tandem WD40 repeats, functions as a docking platform for nuclear receptors, such as the androgen receptor (AR) and estrogen receptor (ER), which bind to the first 70 amino acids of MEP50 (4). This β-propeller architecture also provides a conformational scaffold that enables direct protein–protein interactions with PRMT5, supporting its assembly into the functional methylosome complex (5). In prostate adenocarcinoma, MEP50 stabilizes AR-p53 heterodimers at a subset of androgen-responsive genes, contributing to AR’s control over metastatic prostate cancer cell survival, apoptosis, and proliferation (6). Similarly, in breast cancer models, tamoxifen induces nuclear translocation of the PRMT5/MEP50 methylosome complex, where it catalyzes ERα methylation and promotes the recruitment of corepressors, such as SMRT and HDAC1, leading to the suppression of ERα transcriptional activity (7). However, comprehensive genomic analyses and biophysical studies of both wild-type and mutant MEP50 proteins, encompassing human germline and cancer-associated variants, remain to be done. This is important to understand genes and their function by defining the sequence–structure–molecular dynamics (MD) relationships that give rise to the emergent properties of the encoded protein, namely function and dysfunction. The data described in this study emphasizes the evolutionary conservation of alternatively spliced isoforms of MEP50, which are present across multiple species, suggesting their functional significance. Structural analyses of these isoforms, including homology modeling and MD simulations, reveal key features, such as the seven-bladed WD40 β-propeller architecture, essential for its scaffolding functions. In addition, both isoforms share the conserved region for binding to steroid receptors that regulate endocrine cells. Finally, analysis of conserved amino acids and mutation-induced disruptions provides insights into how structural alterations in MEP50 compromise its stability and impair interactions with key partners, such as the AR and PRMT5. These molecular insights are vital for deciphering the mechanisms by which MEP50 influences hormone-related signaling pathways and contributes to oncogenic processes.

## Materials and methods

### MEP50 knockdown data acquisition and enrichment analysis

Transcriptomic datasets from the Gene Expression Omnibus (GEO) contain RNA-seq profiles of endocrine tumor cell lines subjected to MEP50 perturbation. In particular, we analyzed GSE75743 and GSE154951, representing prostate and breast cancer models (8, 9). We processed each dataset using GEO2R and performed differential expression analysis with DESeq to compare MEP50 knockdown samples to their respective controls (10). We then applied FGSEA to rank genes by their differential expression and identify significantly enriched pathways using the KEGG 2023 database (11, 12).

### MEP50 paralog and ortholog sequence collection, filtering, and analysis

To analyze conserved features among human WD40 domain-containing paralogs, we began with the PF00400 seed alignment, which encompasses 1,465 WD40 repeat sequences (13). This alignment was imported into BioEdit, and sequences were filtered to retain only the 95 annotated human WD40 repeats. A consensus sequence representing these 95 human WD40 domains was generated in BioEdit using the built-in consensus tool (14). This consensus was then aligned to the six annotated WD40 repeats within WDR77 (MEP50) using ClustalW in BioEdit to assess the conservation of canonical WD40 motifs and sequence similarity (15). Ortholog analysis was carried out by collecting all orthologs of MEP50 (WD repeat-containing protein 77) from the current NCBI Orthologs database, yielding a total of 637 protein sequences (16). These sequences were imported into BioEdit, where they were aligned using ClustalW. To ensure consistent comparison, sequences were filtered to retain only those matching the length of the human MEP50 protein (342 amino acids), resulting in a curated set of 200 orthologs. A sequence variability logo was generated using WebLogo 3, and conservation analysis was performed using Jalview (17, 18). Known human post-translational modifications were annotated based on the curated data from PhosphoSitePlus (19). Secondary structure prediction and visualization of the multiple sequence alignment were carried out using ESPript 3.0 (20), incorporating both alignment and structural features to highlight conserved motifs and structural domains across orthologs.

### Phylogenetic reconstruction of the MEP50 short isoform

Thirteen orthologs of the s-MEP50 (278 amino acids) were identified using NCBI Protein BLAST, with the human WDR77 short isoform (NP_001303993) as the query (16). Retrieved sequences were aligned using ClustalW in BioEdit, and a sequence identity matrix was generated to assess the overall conservation (14, 15). To reconstruct evolutionary relationships, we applied the minimum evolution (ME) method in MEGA11 (21). Evolutionary distances were calculated using the Poisson correction model (22), and node support was evaluated with 1,000 bootstrap replicates. The resulting phylogeny recapitulated established mammalian relationships, revealing conserved primate, cetartiodactyla, and carnivoran clades, consistent with the functional constraint on the s-MEP50 across divergent lineages.

### MD-associated parameter measurements

MD simulations were performed to characterize the structural and dynamic effects of MEP50 and its variants (23). Residue-level flexibility was assessed by calculating root mean square fluctuations (RMSFs) from the average structure over the simulation trajectories. Binding free energies were computed throughout the simulations alongside measurements of radius of gyration (Rg) and solvent-accessible surface area (SASA). Non-covalent interactions, including hydrogen bonds, electrostatic, hydrophobic, and other favorable contacts, were continuously monitored to evaluate their contributions to complex stability. The simulations were based on the MEP50:PRMT5 complex structure (7L1G) obtained from the Protein Data Bank (24). Subsequently, we used in silico synthesis to add the N-terminal and C-terminal peptides that were lacking from the MEP50 structure. The s-MEP50 model was derived by homology-based methodology using the structural constraint approach (25). System preparation included structural refinement and energy minimization using the macromolecules and protein design modules within the Discovery Studio. Simulations followed a standard dynamics cascade comprising an initial heating phase of 150 ps, an equilibration phase of 500 ps, and a production phase of 2000 ps. Data were saved at defined intervals throughout each phase (26). Ten independent replicates were conducted per system, each initiated with a distinct random seed to ensure thorough conformational sampling. Mutation energy calculations accounted for pH dependence, and simulations were performed without parallel processing to maintain consistency across runs.

## Results

### Evolutionary genomics identifies residues needed for MEP50 structural integrity and its regulation

**Figure 1:**
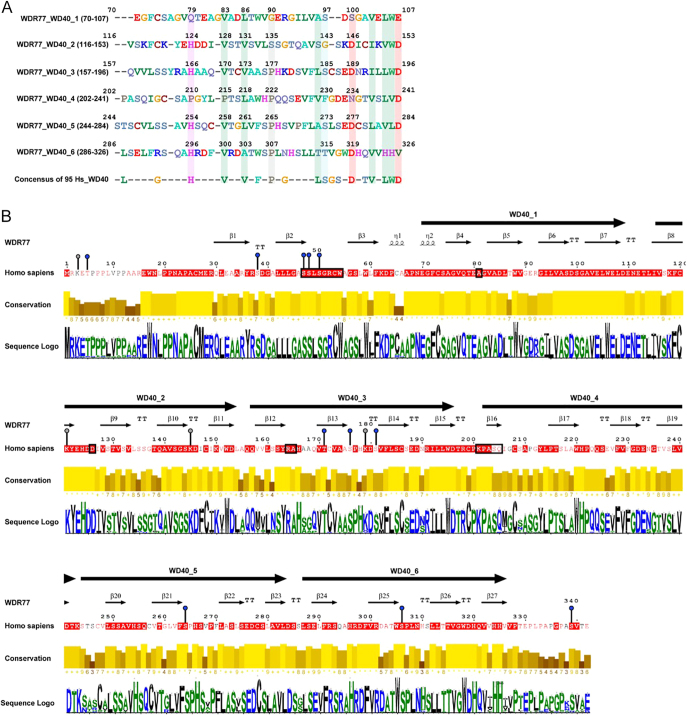
Sequence conservation and structural annotation of MEP50 across human paralogs and evolutionary orthologs. (A) Alignment of 95 human WD40 repeat-containing proteins highlights residues conserved within MEP50 (WDR77) WD40_1–WD40_6 domains. (B) Multiple sequence alignment of MEP50 orthologs across 200 species illustrates conservation along the 342 amino acid full-length isoform. WD40 domains are indicated with large black arrows and annotated; accordingly, secondary structure elements are annotated above the sequence. Post-translational modification sites are marked (blue, phosphorylation; gray, ubiquitination). The black boxes denote regions critical for PRMT5 interaction. The yellow bars indicate per-residue conservation; sequence logos below highlight tolerated variation at less conserved positions.

We analyzed 95 human WD40 domain-containing paralogs to identify conserved residues within the six WD40 repeats of MEP50. This analysis revealed conserved histidine residues in WD40_2 (H124), WD40_3 (H166), WD40_5 (H254), and WD40_6 (H296). A valine residue was conserved across all WD40 repeats except WD40_4, with conserved positions identified at V83 (WD40_1), V128 (WD40_2), V170 (WD40_3), V258 (WD40_5), and V300 (WD40_6). A second hydrophobic residue, located three residues downstream of this valine, was leucine in WD40_1 (L86), WD40_4 (L218), and WD40_5 (L261) and valine in WD40_2 (V131) and WD40_3 (V173), reflecting conservation of side-chain character across repeats. Proline residues were conserved across WD40_3–6: WD40_3 (P177), WD40_4 (P222), WD40_5 (P265), and WD40_6 (P307). Repeat 3 retained an LS motif at residues 184–185. However, WD40 domains WD40_1 and WD40_5 both conserved the serine but contained an alanine instead of the leucine, while WD40_6 contained a threonine, a comparable phosphorylatable residue. The final eight residues of each repeat showed the highest level of conservation across paralogs (Fig. 1A).

Next, we conducted an ortholog analysis of 200 MEP50 sequences from diverse species, all encoding a 342 amino acid protein identical in length to the canonical human isoform. This analysis revealed that the MEP50 protein remains largely conserved across species. Most sequence variations are localized outside the WD40 domains, indicating that these structured repeats maintain strong evolutionary constraint, while more flexible regions tolerate greater divergence. Among these, WD40_1 showed the highest conservation (89%), followed by WD40_6 (75%), WD40_2 (71%), and WD40_3 (70%). WD40_4 and WD40_5 exhibited lower conservation, at 46 and 40%, respectively. At the residue level, methionine (M), tryptophan (W), and tyrosine (Y) were completely conserved. Aspartic acid (D) showed 94.74% conservation, followed by lysine (K, 89%), histidine (H, 82%), and phenylalanine (F, 80%). Glycine (G, 74%) and asparagine (*n*, 71%) were moderately conserved. Hydrophobic residues, such as leucine (L, 66%) and valine (V, 65%), also showed moderate conservation. Alanine (A, 48%) and isoleucine (I, 43%) were the least conserved residues (Fig. 1B). Several residues conserved in the paralog analysis were also highly conserved across orthologs, indicating evolutionary constraint at these positions. These included histidines H124, H166, H254, and H296, and valines V83, V128, V170, V253, V300, V132, and V173. Prolines P176 and P222 were also conserved, although P265 in WD40_5 varied more across species. The LS motif (residues 184–185) remained conserved. The C-terminal region of each WD40 repeat, identified as highly conserved among paralogs, also showed strong conservation across orthologs. Moreover, we identified several functionally relevant regulatory sites with high conservation. A cluster of serine residues (S47, S48, and S50) upstream of WD40_1 remained conserved, suggesting potential regulation via phosphorylation. Interestingly, this highly conserved phosphorylated region spanning S47 to W55 serves as a major binding interface for the PRMT5:MEP50 complex (Table 1) (5). Nearly all residues involved in this interface remained highly conserved across species, with the notable exception of an ‘SQ’ motif at positions S205 and Q206. However, the divergent residue at this site is a threonine, which is interesting, as the ‘S/TQ’ motif is a recognized target for DNA damage kinases, such as ATR and ATM (27, 28). These findings suggest that this species-specific variation should not have a large influence on the role of the PRMT5:MEP50 complex in the DNA damage response, a function previously associated with this complex. Highlighting the importance of this regulatory site, future studies should investigate whether phosphorylation occurs at this site in response to DNA damage, potentially uncovering a novel regulatory mechanism for PRMT5:MEP50 activity. Furthermore, we found conserved ubiquitination sites at K121 and K145 within WD40_2, and at K179 near T171, a highly conserved phosphorylation site, in WD40_3. In contrast, the phosphorylation sites S176 and S181 were more variable in this region. WD40_5 and WD40_6 each contained one conserved phosphorylated site at S264 and S306, respectively, and finally, at the C-terminus, we found one final conserved site at S339 (Fig. 1B). Together, these findings reveal that MEP50 maintains a high degree of sequence conservation across species, particularly within its WD40 domains, underscoring their critical structural and functional roles. Furthermore, the preservation of key residues and regulatory sites across species suggests essential roles in modulating the molecular function of MEP50.

**Figure 2:**
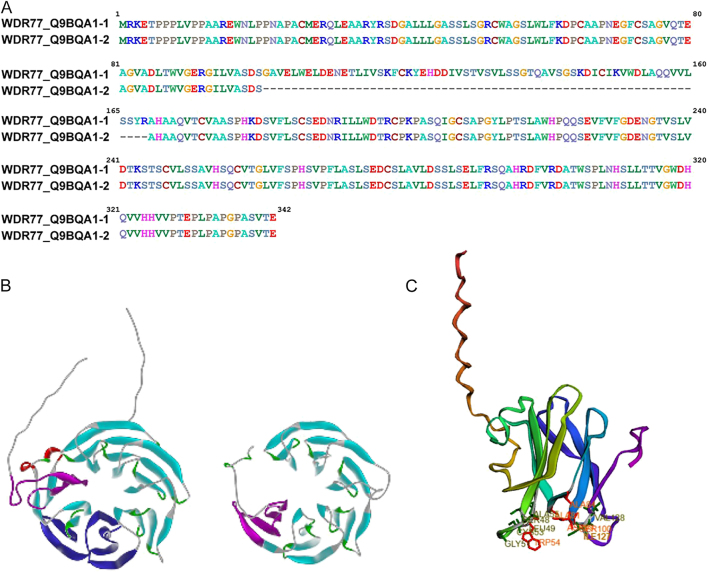
Structural and interaction surface comparison between canonical and short MEP50 isoforms. (A) Pairwise sequence alignment of full-length human MEP50 (WDR77) and the 278 amino acid short isoform (s-MEP50), annotated with UniProt accession IDs. (B) Structural models of canonical MEP50 and s-MEP50 showing WD40 β-propeller architecture and changes in the overall protein structure. (C) Surface patch analysis of both isoforms, mapping predicted protein–protein interaction interfaces.

**Table 1:** Interaction interface between PRMT5 (chain A) and MEP50 (chain B). Residue-level contacts between PRMT5 and MEP50 at the protein–protein interface are categorized by bond type and include electrostatic interactions, hydrogen bonds, and hydrophobic contacts. Each entry specifies the interacting atoms, the bond type, and the interatomic distance in angstroms. Electrostatic and hydrogen bonds predominantly involve residues in the central binding groove of MEP50 (B:47–205) and the PRMT5 interaction surface (A:21–170), while hydrophobic interactions span multiple regions that contribute to complex stabilization.

From residues	To residues	Bond type	Distance
B:LYS201:NZ	A:ASP166:OD2	Electrostatic	3.57246
A:ASN21:HD22	B:ASP126:OD1	Hydrogen bond	1.93406
A:ARG49:HH22	B:SER47:OG	Hydrogen bond	2.41855
A:ARG68:HE	B:ARG52:O	Hydrogen bond	2.47626
A:ARG68:HH21	B:ARG52:O	Hydrogen bond	2.36152
A:ASP165:HN	B:ARG164:O	Hydrogen bond	1.89245
A:GLU169:HN	B:ALA203:O	Hydrogen bond	2.17046
A:ASN170:HD22	B:GLN205:O	Hydrogen bond	1.87691
B:SER50:HG	A:PRO65:O	Hydrogen bond	1.75149
B:CYS53:HG	A:GLU25:OE1	Hydrogen bond	2.41197
B:TRP54:HN	A:PRO24:O	Hydrogen bond	1.85796
B:ARG164:HN	A:ASP165:OD2	Hydrogen bond	2.01046
B:LYS201:HZ3	A:ASP166:OD1	Hydrogen bond	2.04311
B:ALA203:HN	A:ILE167:O	Hydrogen bond	2.22807
B:SER204:HG	A:GLU169:OE1	Hydrogen bond	3.05836
B:GLN205:HN	A:ASN170:OD1	Hydrogen bond	1.77534
A:PRO24:HA	B:TRP54:O	Hydrogen bond	2.46472
A:THR67:HA	B:SER50:OG	Hydrogen bond	2.83833
A:ARG164:HA	B:ARG164:O	Hydrogen bond	3.08918
A:ARG164:HD2	B:ALA165:O	Hydrogen bond	2.62451
A:ILE168:HA	B:ALA203:O	Hydrogen bond	2.40966
B:CYS53:HA	A:PRO24:O	Hydrogen bond	2.30828
B:LYS201:HE2	A:ASP166:O	Hydrogen bond	2.31996
B:SER204:HA	A:ASN170:OD1	Hydrogen bond	2.66058
B:SER204:HB2	A:GLU169:OE1	Hydrogen bond	2.92596
A:ARG68:NH1	B:TRP54	Electrostatic	3.366
A:HIS47	B:TRP54	Hydrophobic	5.27225
A:HIS47	B:TRP54	Hydrophobic	5.20001
B:ALA81	A:CYS22	Hydrophobic	4.51158
B:ALA165	A:ILE167	Hydrophobic	4.69601
B:ALA165	A:ILE168	Hydrophobic	5.06572
B:PRO202	A:ILE167	Hydrophobic	4.76546
B:ALA203	A:ILE168	Hydrophobic	4.53013
A:HIS47	B:LEU49	Hydrophobic	4.39068
A:PHE50	B:LEU49	Hydrophobic	5.28455
B:TRP54	A:ARG68	Hydrophobic	5.11059

### MEP50 undergoes alternative splicing to produce isoforms with distinct structural features and regulatory potential

The most recent NCBI annotation confirms that the MEP50 locus encodes two distinct isoforms, which we refer to here as MEP50 (342 amino acids) and short MEP50 (s-MEP50) (278 amino acids). When searching available MEP50-specific antibodies, we found that some antibodies show two distinct bands. One notable example targets the canonical isoform of MEP50, which runs at ∼43 KDa, but also demonstrates a second band at ∼34 KDa (Supplementary Fig. S1A (see the section on Supplementary materials given at the end of the article), ThermoFisher, USA, PA5-81123). However, all scientific studies to date have only considered the gene transcript encoding a 342 amino acid protein, which corresponds to the larger ∼43 kDa band detected on western blot. Thus, we searched multiple genomes for evidence of a shorter spliced isoform. Indeed, we describe the existence of a 278 amino acid protein with the MW of 29,651 Daltons (Fig. 2A). Our modeling reveals that both the short and long MEP50 isoforms share the first 140 amino acids, encompassing the AR-binding site located within the first 70 residues. However, the biophysical properties of this domain remain to be defined (Fig. 2B). Thus, we began with the MD simulations to investigate the structural importance and regulatory features of the AR-binding domain. To this end, we performed an autopatch analysis, a computational method that automatically identifies and analyzes specific regions on a protein’s surface, called ‘patches,’ which often contain critical functional sites for interactions with other molecules. Interestingly, these analyses identified amino acids mediating protein–protein interactions, specifically residues W54, A81, A84, D99, S100, and I127, highlighting the significance of the first WD40 domain (Fig. 2C). Notably, the PRMT5-binding interface, which mostly interacts with the N-terminal region, particularly residues 47–54, remains largely intact in the s-MEP50, with only D126 and R164 removed (Table 1 and Fig. 2A). However, at the level of the protein structure, this alternative splicing removes the final β-sheet of the first WD40 domain, the entirety of the second WD40 domain, and the first β-sheet of the third WD40 domain. This structural variation suggests that the PRMT5 interaction site may shift within the s-MEP50 to engage with alternative WD40 domains. Given that the major binding region spanning S47 to W54 remains fully intact, PRMT5 likely still binds, albeit potentially with altered affinity. This possibility warrants further structural and biochemical investigations to define the precise impact of isoform-specific truncations on PRMT5:MEP50 complex formation. Another essential feature of this domain is that the three major phosphorylation sites located within the S47 to W54 binding region of PRMT5 remains present in the s-MEP50. Thus, it is likely that both the AR and PRMT5 may compete for this highly phosphorylated binding region, revealing a potential regulatory switch for the molecular functions of MEP50. This region likely serves as a key regulatory hub that coordinates interactions between MEP50, PRMT5, and the AR, with phosphorylation at these residues potentially modulating complex formation, stability, or enzymatic activity.

**Figure 3:**
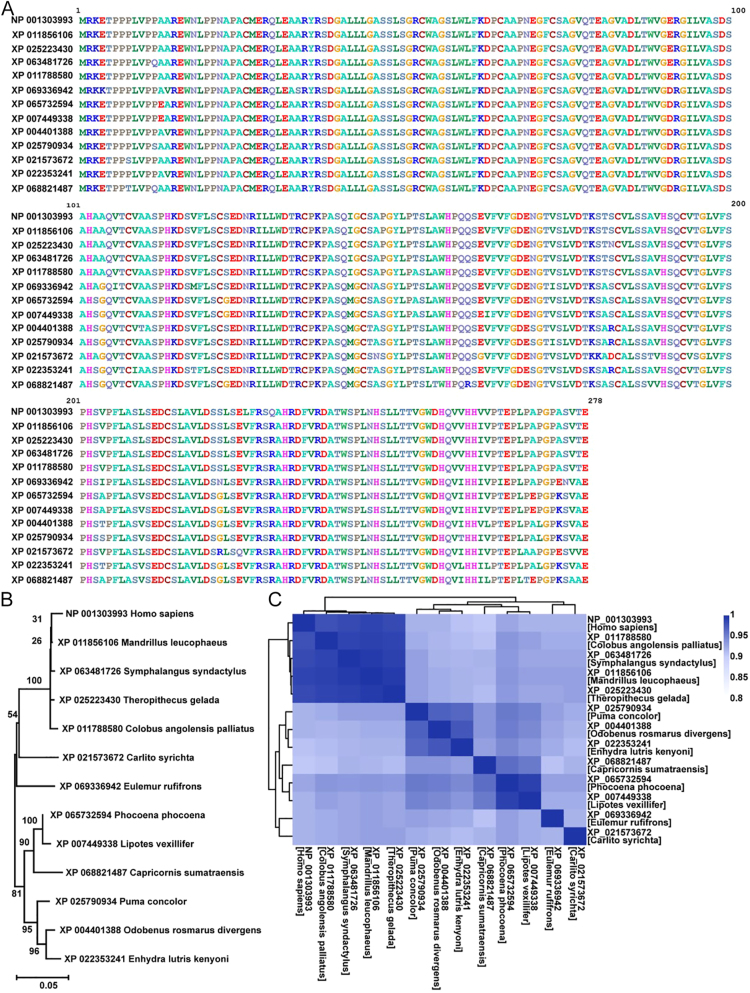
Evolutionary conservation and phylogenetic distribution of s-MEP50 across mammals. (A) Multiple sequence alignment of the 278 amino acid short-MEP50 (s-MEP50) isoform across diverse mammalian species, annotated with NCBI RefSeq accession numbers and species names. (B) Phylogenetic tree of s-MEP50-encoding species, with branch tips labeled by species and corresponding RefSeq accession. (C) Heatmap of per-residue conservation scores across the aligned s-MEP50 sequences.

### Evolutionary analysis demonstrates alternatively spliced isoform is highly conserved across multiple species

We found evidence of this s-MEP50 isoform in a wide range of organisms, suggesting that evolutionary pressure has preserved isoform variation as a constant feature of the *MEP50* gene. Simultaneous searches in Ensembl support the existence of both isoforms. We gathered additional evidence for isoform diversity through evolutionary genomics. Organism-specific database searches identified transcripts in species closely related to *Homo sapiens* that exhibit similar patterns of hormonal regulation. For instance, *Macaca fascicularis* (crab-eating macaque) expresses isoforms of 342, 309, 278, and 318 amino acids in length. Likewise, *Pan troglodytes* (chimpanzee) encodes isoforms of 343, 344, and 298 amino acids, and bonobos express isoforms of 352, 342, 348, 311, and 284 amino acids. Despite this diversity, only the single canonical MEP50 has been considered in humans to date.

**Figure 4:**
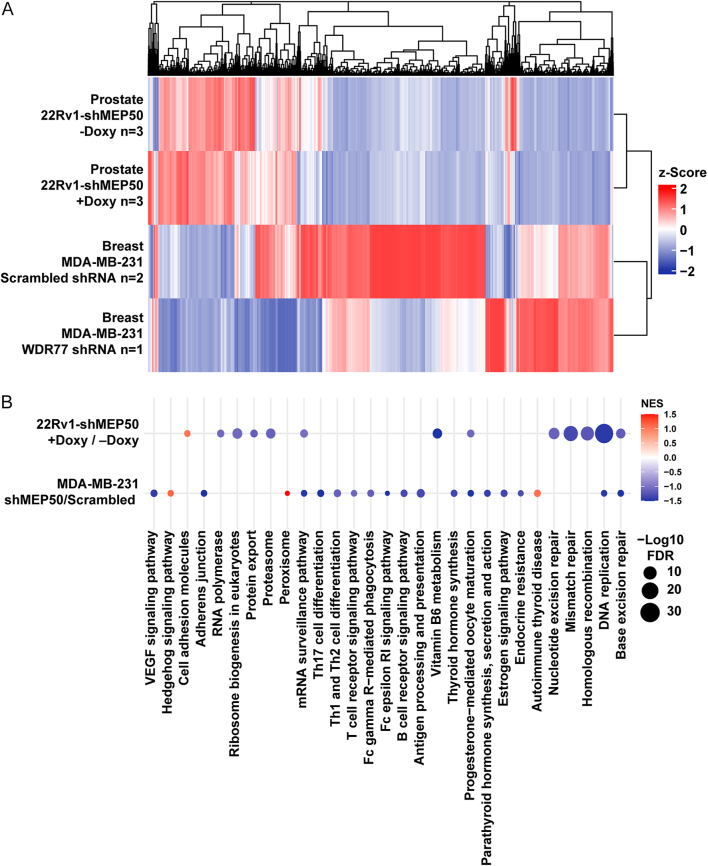
Transcriptomic consequences of MEP50 (WDR77) knockdown in endocrine cancer models. (A) Heatmap of gene expression changes following MEP50 knockdown in prostate and breast cancer cells. Z-scores reflect relative expression changes (red, upregulated; blue, downregulated). Expression profiles reveal distinct, tissue-specific transcriptional responses to MEP50 depletion. (B) Gene set enrichment analysis (GSEA) of RNA-seq data from MEP50-depleted cells. The bubble plot shows normalized enrichment scores (NESs) for significantly altered pathways. Endocrine signaling and DNA damage response pathways are negatively enriched in both models, indicating disruption of hormone-regulated transcription and genome maintenance programs.

To characterize this s-MEP50, we examined its evolutionary context by comparing its sequence to orthologs in other mammals of similar length (278 a.a.) (Fig. 3A). We constructed a phylogenetic tree that illustrates these relationships. The human s-MEP50 clusters closely with sequences from other higher primates, including the mandrill, siamang, gelada, and Angolan colobus monkey. This tight grouping suggests that these primates retain a highly conserved form of the protein, reflecting a shared essential function. In contrast, isoforms from the tarsier and lemur diverge from the higher primate cluster, indicating a distinct evolutionary path. At the opposite end of the main branch, the tree reveals a relationship between the human isoform and those found in aquatic mammals (Fig. 3B). The porpoise and Yangtze River dolphin, closely grouped with the Sumatran serow, share several sequence features with the human protein. Within the carnivore branch, the puma shows a slightly divergent sequence compared to the more similar sea otters and walrus (Fig. 3B). To further contextualize these findings, we analyzed a sequence similarity matrix. The human s-MEP50 shares over 98% identity with other higher primates, such as mandrill (98%), gelada (98%), siamang (98%), and Angolan colobus monkey (98%), which aligns with the phylogenetic clustering. Isoforms from the tarsier and lemur show ∼92% identity, consistent with their phylogenetic distance (Fig. 3C). Aquatic mammals also exhibit high similarity to the human isoform, with values of 92% for porpoise and 92% for the Yangtze River dolphin. In the carnivore group, the walrus and sea otter are highly like one another (97%) and to the puma (96%), although the puma shows a slightly reduced similarity to the human sequence (92%) (Fig. 3C). Together, our results highlight a deeply conserved and functionally relevant s-MEP50, suggesting that its evolutionary persistence reflects essential roles maintained across diverse mammals.

### Cancer-associated mutations in MEP50 disrupt conserved bonding networks and induce widespread structural destabilization

To investigate the clinical relevance of MEP50, we analyzed large-scale cancer genomics data from cBioPortal. We found that MEP50 is altered in multiple cancer types through point mutations and deep deletions. Notably, in endocrine-related cancers, such as breast cancer, ovarian epithelial tumors, endometrial cancer, adrenocortical carcinoma, and prostate cancer, we find frequent amplification of the MEP50 locus (Supplementary Fig. S1B). These alterations suggest that MEP50 activity may contribute to the pathogenesis of hormone-driven tumors. Moreover, MEP50 alterations frequently co-occurred with changes in other components of the methylosome complex, including PRMT5, CLNS1A, RIOK1, and COPRS, indicating a potential coordinated dysregulation of this epigenetic machinery (Supplementary Fig. S1C). For this reason, we next focused on understanding how specific residues influence the structural and dynamic properties of MEP50 that may result in its dysregulation. We selected two core tryptophan residues (W59 and W152), essential for maintaining the β-propeller fold, as positive controls which we predict to cause significant local disruptions, alongside three endocrine cancer-associated mutations: A97T, E107K, and A303V. To dissect their functional roles, we evaluated how each variant influences the stability of the β-propeller architecture. Notably, A303 was the only mutated residue not highly conserved across MEP50 orthologs (Table 2).

**Table 2:** Local interaction profiles of conserved and cancer-associated MEP50 mutations. Change in folding free energy (ΔΔG, kcal/mol) for MEP50 mutants, as calculated by structural modeling. W59R and W152K affect conserved tryptophan residues critical for WD40 domain integrity, while A97T, E107K, and A303V correspond to somatic mutations found in endocrine cancers.

Mutation	Energy	Effect
W59R	5.66	Destabilizing
W152K	6.2	Destabilizing
A97T	−1.06	Destabilizing
E107K	1.97	Destabilizing
A303V	1.57	Destabilizing

To define the structural mechanisms of conserved tryptophan residues in MEP50, we first examined the non-covalent interaction formed by W59 and W152. In its native state, W59 forms a dense network of stabilizing interactions, including hydrogen bonds, with L44 and S57, π-sulfur contact with C73, and multiple π-alkyl interactions with M7, A23, and P24. Substituting W59 with arginine disrupts these interactions, preserving only a few hydrogen and alkyl bonds while eliminating π-alkyl and π-sulfur contacts (Supplementary Table 1). Likewise, wild-type W152 forms multiple stabilizing interactions, including hydrogen and carbon–hydrogen bonds, with A140, Q139, and A158, and π-alkyl contacts with V103, V150, and V159. In the W152K mutant, lysine abolishes the aromatic interactions and diminishes the hydrogen bonding network. These results highlight the structural importance of W59 and W152 in stabilizing the WD40_1 and WD40_2 repeats, as each tryptophan anchors a network of aromatic, hydrophobic, and hydrogen bond interactions that maintain the local fold. Replacement of W59 with arginine or W152 with a lysine, which are polar, non-aromatic residues, removes the key π-mediated contacts and introduces steric and electrostatic strain. Although chemically distinct, both substitutions disrupt local packing, emphasizing the essential role of tryptophan in preserving WD40 repeat integrity. To explore the structural and biological relevance of mutations found in endocrine cancers, we next analyzed patient-derived variants by examining the non-covalent interaction networks of wild-type and mutant residues, with a focus on their impact on WD40 domain architecture. We first looked at the cancer mutation A97T, which was identified in a patient with endometrioid carcinoma. A97 forms modest hydrogen and alkyl interactions in the wild-type state. Substituting the alanine to a threonine preserves hydrogen bonding but introduces an additional side-chain interaction. In contrast, E107K, a variant found in pancreatic tumors, forms a critical salt bridge with K118, engaging in multiple hydrogen bonds that are disrupted upon substitution for lysine. The E107K variant replaces the electrostatic network with weaker hydrophobic contacts, reducing the local structural cohesion. Finally, we reveal that the A303V variant found in endometrioid carcinoma contributes to local hydrophobic packing through hydrogen bonds with N302 and T314 and alkyl contacts with L274 and L313. The A303V mutation preserves these interactions but introduces additional contacts with V262 and A272.

We extended this analysis to evaluate how endocrine tumor-associated mutations alter the global flexibility of MEP50. To do this, we calculated residue-level root mean square fluctuations (RMSFs) for all currently known endocrine-related cancer variants and compared the results to the wild-type structure (Supplementary Table 2). Each mutation produced a unique pattern of flexibility changes, which we mapped to the six WD40 repeats of the β-propeller fold. The A303V mutation, located in WD40_6, increased local flexibility at the mutation site (RMSF Δ: +0.43 Å at A303) and elevated fluctuations across WD40_6 (+0.055 Å). This mutation also increased RMSF in WD40_2 (+0.057 Å), WD40_4 (+0.063 Å), and to a lesser extent WD40_5 (+0.035 Å), indicating that changes originating in the C-terminal repeat propagated allosterically to central regions of the structure. The A97T variant, positioned in WD40_1, maintained local flexibility at the mutation site but redistributed motion across the structure. This variant increased RMSF in WD40_4 (+0.008 Å), WD40_6 (+0.114 Å), and slightly in WD40_5 and WD40_2, highlighting a dynamic shift from the N-terminal to C-terminal half of the protein. The E107K mutation, also in WD40_1, disrupted a conserved salt bridge and increased flexibility across several domains. We recorded marked RMSF gains in WD40_4 (+0.078 Å), WD40_5 (+0.071 Å), and WD40_6 (+0.086 Å). These findings suggest that the loss of electrostatic stability in WD40_1 weakens inter-repeat communication and destabilizes distal regions. The D146H substitution, located between WD40_2 and WD40_3, induced widespread rigidity. RMSF values decreased in WD40_6 (−0.242 Å), WD40_1 (−0.046 Å), WD40_2 (−0.044 Å), and WD40_3 (−0.038 Å), suggesting that this variant reinforces inter-repeat packing at the cost of overall flexibility. G76R, which resides in WD40_1, reduced flexibility throughout the structure. The mutation introduced a bulky charged residue that rigidified WD40_6 (−0.034 Å), WD40_5 (−0.027 Å), and modestly dampened motion in other repeats. These reductions indicate that steric hindrance in WD40_1 propagates globally and suppresses backbone fluctuations. L86P, another WD40_1 mutation, produced a similar effect. This substitution slightly decreased flexibility in WD40_5 (−0.015 Å), WD40_6 (−0.017 Å), and the core of WD40_2 and WD40_3, reinforcing the conclusion that alterations in repeat 1 influence the entire β-propeller. The S98Y mutation, also in WD40_1, further confirmed this pattern. Although local flexibility remained unchanged, we measured reduced RMSF values in WD40_5 (−0.043 Å) and WD40_6 (−0.021 Å), suggesting that loss of polar contacts or π-interactions at this site dampens distant dynamic zones. T79M, which lies in WD40_1, produced the opposite effect. This mutation increased flexibility in WD40_4 (+0.025 Å), WD40_5 (+0.010 Å), and especially WD40_6 (+0.108 Å), indicating that even conservative substitutions can amplify backbone mobility across structurally distant repeats. Y163N, which resides in WD40_2, exerted more subtle effects. It slightly increased flexibility in WD40_3 (+0.008 Å) while reducing motion in other regions. These shifts suggest that this mutation produces a more localized redistribution of motion without significant structural destabilization. V182M, located in WD40_3, reduced RMSF values across the structure. This mutation decreased flexibility in WD40_5 (−0.037 Å) and WD40_6 (−0.026 Å), pointing to a restrained dynamic state induced by hydrophobic overpacking.

Together, these findings demonstrate that mutations in MEP50 affect protein dynamics in both a site-specific and repeat-wide manner. Substitutions in WD40_1 often act as hubs for allosteric regulation, transmitting structural strain or dampening through multiple repeats. Mutations in the C-terminal region, such as A303V, propagate motion back toward the core. These distinct dynamic signatures highlight the structural fragility of the β-propeller architecture and support a model in which cancer-associated mutations compromise MEP50 function through coordinated disruption of inter-repeat dynamics.

### Molecular characterization of MEP50 reveals its impact on cancer-related signaling

To complement our structural and MD analyses and gain functional insights, we next investigated the transcriptional consequences of MEP50 dysregulation in endocrine cancer model systems. By leveraging RNA sequencing data from genetic knockdown experiments in cancer-derived cell lines of prostate and breast origin (8, 9), we aimed to elucidate how MEP50 depletion reshapes gene expression programs relevant to tumor biology. This approach linked molecular alterations in MEP50 to context-specific transcriptional changes, revealing that its genetic depletion triggers divergent transcriptional responses across hormone-driven cancer models, consistent with tissue-dependent functions (Fig. 4A). To understand the molecular consequences of MEP50 knockdown across cancer-derived cell models, we performed gene set enrichment analysis (GSEA).

GSEA revealed that, despite distinct transcriptional profiles, MEP50 knockdown induces overlapping molecular programs across cancer models, prominently affecting endocrine signaling, DNA repair, and protein homeostasis pathways. Both the breast and prostate cancer models exhibit significantly reduced enrichment of endocrine-related gene sets involved in progesterone-mediated regulation (breast, NES = −1.68; prostate, NES = −1.06). In the breast cancer model, MEP50 loss further diminishes the expression of genes associated with endocrine resistance (NES = −1.28), estrogen signaling (NES = −1.34), and thyroid hormone activity (NES = −1.41) (Fig. 4B). MEP50 depletion also suppresses DNA repair programs in both models, with downregulation of genes involved in DNA replication (breast, NES = −1.45; prostate, NES = −1.46) and base excision repair (breast, NES = −1.56; prostate, NES = −1.17). Notably, prostate cancer cells demonstrate additional downregulation of mismatch repair (NES = −1.33), homologous recombination (NES = −1.21), and nucleotide excision repair pathways (NES = −1.12), implicating a more critical role for MEP50 in maintaining genome integrity in this context (Fig. 4B). Similarly, both models show decreased expression of genes involved in the mRNA surveillance pathway (breast, NES = −1.46; prostate, NES = −1.02), indicating disruption of protein homeostasis. In addition, prostate cancer cells exhibit reductions in the expression of genes linked to the proteasome (NES = −1.12), protein export (NES = −1.15), ribosome biogenesis (NES = −1.07), and RNA polymerase (NES = −1.06), reinforcing a broader impairment in proteostasis machinery. Finally, MEP50 knockdown in the breast cancer model suppresses immune-related gene sets, including those regulating immune cell differentiation and receptor signaling, a response not mirrored in prostate cancer cells. Together, these findings demonstrate that MEP50 depletion disrupts core regulatory programs in a tissue-specific manner, with shared effects on endocrine signaling, DNA repair, and proteostasis, while revealing distinct transcriptional vulnerabilities shaped by endocrine lineage.

## Discussion

This study defines the genomic, structural, and functional landscape of MEP50, a WD40 domain scaffold central to hormone signaling and endocrine cancers. By integrating evolutionary genomics and molecular modeling, we discover a conserved alternative splicing event in MEP50 that removes the last β-sheet of the first WD40 domain, the entire second WD40 domain, and the first β-sheet of the third WD40 domain. Our molecular modeling of cancer-associated mutations combined with transcriptomic profiling demonstrates that the β-propeller architecture of MEP50 is structurally constrained and essential for function. Disruption of this architecture by mutation leads to dysfunction. We further examine the molecular and functional consequences of MEP50 loss, showing its impact on hormone signaling, DNA repair, and cellular homeostasis in endocrine cancer contexts.

WD40 domains, which form highly stable seven-bladed β-propeller scaffolds, rank among the most abundant protein domains in eukaryotes and emerged during the early stages of eukaryotic evolution. These domains mediate diverse protein–protein interactions, including scaffolding and the cooperative assembly of dynamic multi-subunit complexes involved in chromatin regulation and signal transduction (29). The WD40 β-propeller is functionally indispensable for the PRMT5:MEP50 complex, with six canonical blades and a truncated seventh blade forming a rigid scaffold that enables PRMT5 interaction and appropriate substrate positioning (30). While two bands for MEP50 were observed by western blot in the original publication (2) and several antibodies have seen both the expected and s-MEP50, our evidence further supports the existence of this short isoform. Therefore, we are optimistic that future biochemical, biophysical, and cellular biological studies will build on this observation to achieve functional discoveries. In particular, this isoform removes large segments of the WD40 repeat architecture, including entire blades and interconnecting β-strands that are evolutionarily retained in the canonical form. These missing elements overlap with residues essential for PRMT5 interaction surfaces, predicting a theoretical shift in affinities and structural scaffolding with PRMT5, but not AR binding, which needs to be further explored. Through ortholog analysis of the canonical isoform, we found that amino acids mediating the interface with PRMT5 are highly conserved. Notably, our data highlight a potential regulatory region involving phosphorylation at three serine residues, namely S47, S49 and S50, and a putative DNA damage-responsive site at S204. Phosphosite analysis predicts that the serine cluster at positions 47–50 is a candidate substrate for ERK7, a member of the CMGC family of MAP kinases (19). Notably, human ERK7 is predicted to be highly expressed in endocrine tissues, such as the pancreas (31). In support of its potential relevance to endocrine-related cancers, ERK7 has been shown to downregulate AR and glucocorticoid receptor transcriptional activity in human bronchial epithelial cells through its interaction with Hic-5, a shared co-regulator of both AR and glucocorticoid receptor (32). If MEP50 phosphorylation at S47–50 is mediated by ERK7, this could represent a context-specific mechanism for modulating methylosome activity in hormone-responsive cancers. Such a mechanism may provide an additional layer of regulatory control over PRMT5-dependent signaling in endocrine tumor contexts, linking upstream kinase activity to chromatin-associated transcriptional programs. Moreover, the S204 ‘SQ’ motif, a canonical recognition sequence for ATM/ATR kinases, suggests that MEP50 may also be subject to regulation in response to genotoxic stress. Previous studies in breast cancer cells have shown that PRMT5 activity supports DNA repair by promoting BRCA1 mRNA stability through m6A demethylation pathways (33). If MEP50 phosphorylation at S204 regulates its interaction with PRMT5 under DNA damage conditions, this modification may act as a molecular switch to redirect methylosome activity toward DNA repair gene networks.

Our integrated structural and transcriptomic analyses establish MEP50 (WDR77) as a critical driver of oncogenic programs in endocrine tumors. By maintaining the structural integrity of the PRMT5 methylosome, MEP50 governs downstream signaling pathways by facilitating the accurate substrate positioning for methylation. Our analysis of large-scale cancer genomics data reveals that MEP50 frequently undergoes amplification and point mutation in hormone-driven cancers (34, 35). In many cases, we find that these alterations co-occurred with mutations in other methylosome components, including PRMT5, CLNS1A, RIOK1, and COPRS. This pattern suggests a coordinated disruption of the epigenetic machinery required for PRMT5 activity, in line with previous findings that PRMT5:MEP50 complexes are upregulated across diverse tumors and contribute to proliferative signaling (36). Our structural modeling reveals that specific cancer-associated mutations destabilize MEP50’s highly conserved WD40 β-propeller. Variants, such as E107K, disrupt a conserved salt bridge and weaken the inter-blade packing, while mutations, such as A303V, although not highly conserved across evolution, produce allosteric effects that extend beyond their local environment in WD40_6. MD simulations show that these cancer mutations induce distinct structural outcomes that will likely disrupt the PRMT5:MEP50 interaction. Overall, our MD simulations indicate that missense mutations disrupt both local and global protein dynamics, likely impairing MEP50’s scaffolding function and interfering with the correct positioning of PRMT5 substrates for symmetric dimethylation of arginine.

To explore the potential functional consequences of MEP50 disruption in hormone-responsive cancers, we analyzed RNA-seq data from MEP50 knockdown experiments in breast and prostate cancer models (8, 9). Despite tissue-specific differences, both models showed downregulation of endocrine signaling and DNA repair pathways. These findings raise the possibility that the cancer-associated mutations, which we find impair the scaffolding interaction of MEP50 with PRMT5, may disrupt methylosome function and contribute to similar transcriptional dysregulation in tumors. Indeed, PRMT5’s function in endocrine contexts further supports this model. In prostate cancer, PRMT5 catalyzes H4R3 methylation at the AR promoter to activate AR transcription (9). This activity is facilitated by pICln, which promotes substrate recognition of histone H4 (9). In breast cancer, tamoxifen treatment induces PRMT5 nuclear translocation, enabling methylation of ERα. This event is a prerequisite for ERα corepressor binding by SMRT and HDAC1 and repression of ERα transcriptional activity (7). PRMT5 also influences DNA repair in the context of endocrine treatment. Following doxorubicin treatment of breast cancer cells, PRMT5 induces nuclear translocation of the RNA demethylase ALKBH5, which removes m6A methylation from BRCA1 mRNA, stabilizing the transcript and enhancing DNA repair capacity to ultimately attenuate doxorubicin efficacy (33). Taken together, our findings support a model in which MEP50 serves as a central scaffolding regulator of methylosome function with important implications for endocrine tumor biology. The cancer-associated mutations evaluated in this study destabilize MEP50, likely impacting methylosome complex integrity and PRMT5 enzymatic activity, thereby disrupting hormone signaling and DNA repair pathways.

In conclusion, our comprehensive analyses establish MEP50 as an evolutionarily conserved scaffold essential for maintaining the integrity and regulatory capacity of the PRMT5 methylosome in endocrine tissues. Alongside the structural and functional consequences of cancer-associated mutations, we define a conserved alternatively spliced isoform of MEP50 that removes the essential elements of the WD40 β-propeller. This variant is predicted to alter scaffolding interactions with PRMT5 while retaining AR binding, suggesting it may act in a context-dependent or PRMT5-independent manner. Its evolutionary conservation implies regulatory significance, and future studies delineating its expression dynamics, protein stability, and binding partners will be essential for determining whether s-MEP50 serves as a dominant-negative or supports alternative MEP50 functions. Furthermore, through ortholog conservation and cancer-associated mutations, we identify a network of critical residues and structural domains required for MEP50 stability, along with candidate sites for post-translational regulation. Disruption of this architecture is likely to compromise methylosome catalytic activity, leading to broad dysregulation in hormone signaling, DNA repair, and cellular homeostasis. These findings position MEP50 as a central vulnerability in hormone-driven cancers and support its development as both a biomarker and therapeutic target in tumors dependent on methylosome function.

## Supplementary materials







## Declaration of interest

The authors declare that there is no conflict of interest that could be perceived as prejudicing the impartiality of the work reported.

## Funding

This work was supported by NIHhttps://doi.org/10.13039/100000002 (grant numbers R01DK52913 (to RU and GL) and R01CA247898 (to GL)); Advancing a Healthier Wisconsin Endowment (to RU); Advancing a Healthier Wisconsin MCW Post-Doctoral Researcher Seed Grant (to GP); the Linda T and John A Mellowes Endowed Innovation and Discovery Fund (to RU); The Joel and Arlene Lee Endowed Chair (to GL); and Markus Family Funds for Discovery and Innovation Family Funds to the Mellowes Center.
