# Supplementary figures and images for: Genomic features of the methylosome protein MEP50 and its implications in hormone signaling and cancer
Source: Endocr Connect. 2025 Sep 24;14(9):e250444. doi: 10.1530/EC-25-0444 (PMC12474799; doi:10.1530/EC-25-0444)

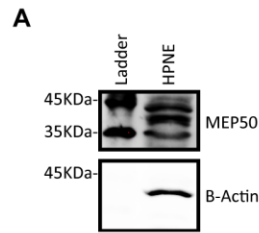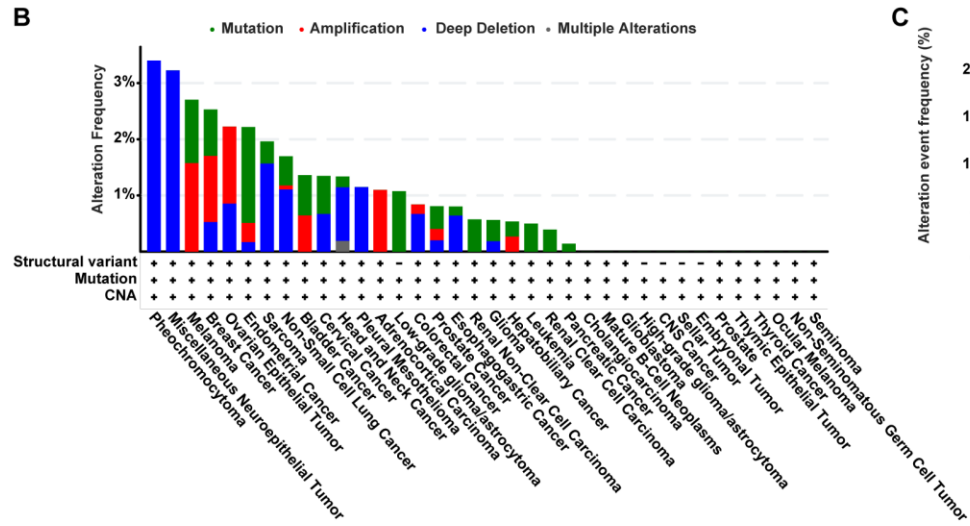

Supplement: Supplementary file 1 [file supplementary_figure_S1.pdf]
